# Analysis on the Development of Digital Economy in Guangdong Province Based on Improved Entropy Method and Multivariate Statistical Analysis

**DOI:** 10.3390/e22121441

**Published:** 2020-12-20

**Authors:** Xue Deng, Yuying Liu, Ye Xiong

**Affiliations:** School of Mathematics, South China University of Technology, Guangzhou 510640, China; 18785609808@163.com (Y.L.); workyep@163.com (Y.X.)

**Keywords:** digital economy, indicator system, improved entropy method, principal component analysis, factor analysis

## Abstract

The lack of adequate indicators in the research of digital economy may lead to the shortage of data support on decision making for governments. To solve this problem, first we establish a digital economy indicator evaluation system by dividing the digital economy into four types: “basic type”, “technology type”, “integration type” and “service type” and select 5 indicators for each type. On this basis, the weight of each indicator is calculated to find the deficiencies in the development of some digital economic fields by the improved entropy method. By drawing on the empowerment idea of Analytic Hierarchy Process, the improved entropy method firstly compares the difference coefficient of indicators in pairs and maps the comparison results to the scales 1–9. Then, the judgment matrix is constructed based on the information entropy, which can solve as much as possible the problem that the difference among the weight of each indicator is too large in traditional entropy method. The results indicate that: the development of digital economy in Guangdong Province was relatively balanced from 2015 to 2018 and will be better in the future while the development of rural e-commerce in Guangdong Province is relatively backward, and there is an obvious digital gap between urban and rural areas. Next we extract two new variables respectively to replace the 20 indicators we select through principal component analysis and factor analysis methods in multivariate statistical analysis, which can retain the original information to the greatest extent and provide convenience for further research in the future. Finally, we and provide constructive comments of digital economy in Guangdong Province from 2015 to 2018.

## 1. Introduction

Digitalization has set the stage for a stream of radical innovations that have the potential to trigger a new technological revolution and cause deep structural changes, which has been increasingly integrated into the economy and society. Digital economy has been proposed to be a kind of brand-new economic form promoting the upgrading of traditional industries and the rapid development of emerging industries, which has had a profound influence on the digital transformation of enterprises.

Digital economy plays a significant role in global economy, has attracted more and more attention among all countries in the trend of digitalization, affecting many aspects of the society such as manufacturing, electronic information industry, artificial intelligence, and public policy. As for the definition of digital economy, many scholars in China and other countries have different opinions. Some American scholars define the digital economy as the sum of the measurable e-commerce and information technology industries [1]. Aguila et al. [2] consider that the digital economy is an economic sector that includes goods and services, whose development, manufacturing, merchandising or supply depends on critical digital technologies, which can be conceptualized into four different subsectors: infrastructure and applications, electronic commerce and new intermediaries. Li [3] believes that digital economy is an economic form that mainly produces in the way of digital technology. Among the definitions of digital economy, the most representative one comes from the G20 Digital Economy Development and Cooperation Initiative released at the G20 Hangzhou Summit in 2016: digital economy refers to a series of economic activities in which the digital knowledge and information is the key factor of production, the modern information network is regarded as the important carrier, and the effective use of information and communication technology (ICT) is the important driving force for efficiency improvement and economic structure optimization. On this basis, more and more scholars have studied the impact of digital economy on various aspects in regions or countries through different ways, and offered their constructive suggestions from different perspectives. Li et al. [4] assessed how new value chains are transforming country-level involvement in worldwide manufacturing and concluded that the digital economy in Asian nations involved revamping business processes through technology innovation, government policies for growth, and digital entrepreneurship. In Amuso’s opinion [5], despite the increasing speed of digital innovation, governments should invest in education and life programs to fully reap the benefits of the digital economy. Schweighofer et al. [6] studied the relations between the digital economy and technology enhanced learning which are hardly investigated. Chen [7] thought that the digital economy has substantially reduced market frictions but also posed new challenges for the efficient functioning of markets and discussed how well-designed policies on competition, regulation, IP protection, and consumer privacy can improve market performance in the digital economy. Based on qualitative in-depth interview with many experts in various fields, Malisuwan et al. [8] used the results of qualitative analyses to assist policy makers in developing strategy and framework of the Thailand’s Digital Economy Plan to foster social and economic benefits in the digital economic era.

In addition, it’s necessary for us to take advantage of different indicators to investigate and evaluate the development of the economy and other fields adequately. Based on this, some scholars have made relevant studies from different perspectives. Strohmaier et al. [9] introduced comprehensive indicators into a new framework for analysis, so as to study the socio-economic system of a country over time. Szeles et al. [10] studied the progress made in digitization and digital economic growth in the EU region by analyzing a certain number of selected specific indicators based on the data of Eurostat from 2001 to 2016, and finally found the policy measures that can promote digital economic growth in the EU regions. Milosevic et al. [11] created a multivariate indicator that can serve as a measurement of digital economic performances by using the Composite I-distance Indicator (CIDI) methodology and evaluated and ranked 28 countries in EU (EU-28) based on their digital performances. Jaime et al. [12] showed a number of indicators to measure economic efficiency in terms of circular economy (CE). Ahmadi et al. [13] focused on modeling economic growth with indicators of knowledge based economy (KBE) introduced by World Bank for a case study in Iran during 1993–2013. Cizmesija et al. [14] formed the new liquidity indicator by using factor analysis and applied econometric models in order to investigate the forecasting properties of the new business survey liquidity indicator, when predicting the direction of changes in Croatian industrial production. Chen [15] built a dynamic indicator to evaluate the ecological economic transition in Chinese provinces since the reform was based on slacks-based measure (SBM) mechanism.

As a matter of fact, some researchers have also made quantitative analyses on their fields by the method similar to us. Bui et al. [16] evaluated 273 keywords and 22 indicators obtained based on the experts’ advice by entropy weight method, fuzzy decision-making trial and evaluation laboratory in supply chains. Huang et al. [17] used two historical data-driven weight calculation approaches including Entropy Weight Method (EWM) and Scatter Degree Method (SDM) to solve the risk assessment of railway dangerous goods transportation system (RDGTS). Li et al. [18] applied principal component analysis (PCA), entropy method and random forest to calculate weighted coefficients of key metrics when assessing the ecological health of large rivers. But references [16,17,18] just used the traditional entropy method and didn’t improve the algorithm.

In this study, in order to evaluate the development scale of digital economy and find the deficiency in the development in Guangdong province, the weight of each indicator selected under the digital economy indicator evaluation system is calculated by the improved entropy method, which can solve as much as possible the problem that the difference among the weight of each indicator is too large in traditional entropy method. Then we took advantage of principal component analysis and factor analysis methods to make a dimension reduction for 20 indicators to prepare for the further study. At the same time, we discussed the change of the scores of digital economic scales in Guangdong Province with the change of time. Finally, we put forward some proposals for the optimization of economic development path on the basis of real data analysis.

The rest of the paper is structured as follows: Section 2 shows the definition of Shannon entropy. Next, we establish the digital economy indicator evaluation system by selecting relevant indicators based on different economic types in Section 3. Then, in Section 4, we introduce the traditional entropy method, the improved entropy method and their concrete calculation steps respectively. Moreover, the comparison between them is presented. In Section 5, the principal component analysis and factor analysis mathematical models are constructed. We conduct an empirical analysis of the specific examples and get the corresponding results by the improved entropy method, principal component analysis and factor analysis to propose some advice for the digital economy of Guangdong Province in Section 6. Finally, in Section 7 we draw some conclusions based on the work done in this article.

## 2. Preliminaries

### Shannon Entropy

In 1948, “Shannon entropy” was firstly put forward to describe the degree of uncertainty in the value of discrete random variables and solve the problem of quantitative measurement of information by Shannon [19]. The definition given by Shannon indicates that the entropy value increases as the uncertainty of the random variable increases, and vice versa. According to the characteristics of Shannon entropy, we can not only measure the randomness and disorder degree of an event, but also judge the dispersion degree of the indicators on the comprehensive evaluation system by entropy value.

For any random variable X, the Shannon entropy is defined as follows:

(1) $$H(X)=-\sum_{i=1}^{n}p_{i}\log_{2}p_{i}$$

where n represents the number of results of X, each result corresponds to a discrete possibility of $p_{i}(p_{i}>0)$ and $\sum_{i=1}^{n}p_{i}=1$.

## 3. Establishment of Indicator Evaluation System

In this section, in order to have a further study on the development state of digital economy in Guangdong Province quantitatively, we constructed the digital economy indicator evaluation system by selecting relevant digital economy indicators. After processing the relevant indicator values in Guangdong Province from 2015 to 2018, the improved entropy method was introduced to calculate the weight of each indicator and reduce the influence of some discrete data. 

“China Digital Economic Development Index in 2017” released by CCID Consulting divided the digital economy into five types: basic-type, resource-type, technology-type, integration-type and service-type. Combined with the indicator system in reference [20], we selected 5 available and effective indicators for each type respectively and explained the reason why we chose them. The indicators we selected are shown in Table 1:

According to Table 1, basic-type digital economy indicators involve two aspects of basic telecommunication and network. Five indicators: length of optical cable line, telephone penetration rates, the number of Internet broadband users, the number of websites and the number of domain names are selected to measure the popularity and development of telecommunications and the Internet.

From the perspective of digital technology, some emerging industries like block chain, big data and artificial intelligence can become members of the technology-type digital economy indicators. However, in view of the difficulties in obtaining their concrete data, we mainly take advantage of information technology industry to measure the development of digital technology. Therefore, IT service revenue, embedded system software revenue, total telecom services, software business income, social fixed asset investment in information transmission, computer service and software industry are used as the technology-type digital economy indicators.

Integration-type digital economy indicators focus on the combination of digital economy, industry and agriculture, aiming to evaluate the application degree of informatization in industry, agriculture and enterprise. Therefore we choose the increase of rural e-commerce comprehensive demonstration counties, the number of designed size enterprises P & D projects, the proportion of enterprises with e-commerce transaction activities, the number of enterprises with informatization and the number of enterprises integrated with industrialization and informatization as integration-type digital economy indicators.

Based on the degree of integration between digital economy and service industry, the service-type digital economy plays an important role in people’s life, learning, entertainment and research. Therefore, we adopt the following indicators: e-commerce turnover, the number of public information on government websites, the number of terminals in electronic reading room of public library, the number of digital TV users, social fixed asset investment in scientific research, technical services and geological survey.

## 4. Improved Entropy Method

### 4.1. Traditonal Entropy Method and Concrete Calculation Steps

Entropy method is one of the objective weighting methods, which determines the weight of each indicator according to the amount of information provided by the observed values. And it is used to judge the discrete degree of indicators, the greater the discrete degree, the greater the influence of this indicator on the comprehensive evaluation [21].

We can figure out the weight of the digital economy indicators by the following steps based on the traditional entropy method:

**Step 1.** Firstly the data should be standardized to eliminate the influence of different dimensions. All indicators are positive in the digital economy evaluation indicator system of Guangdong Province in this paper, which can be standardized according to Formula (2):

(2) $$X_{ij}^{*}=\frac{X_{ij}-\min\{X_{j}\}}{\max\{X_{j}\}-\min\{X_{j}\}},$$

where $X_{ij}$ represents the value of the j−th indicator in the i−th year. i=1,2,⋅⋅⋅,n, j=1,2,⋅⋅⋅,m.n represents the number of years and m represents the number of indicators. 

**Step 2.** Calculate the weight of the j−th indicator in the i−th year:

(3) $$q_{ij}=\frac{X_{ij}^{*}}{\sum_{i=1}^{n}X_{ij}^{*}}.$$

**Step 3.** Calculate the information entropy:

(4) $$e_{j}=-k\sum_{i=1}^{n}\left(q_{ij}\times \ln q_{ij}\right).$$

Generally, $k=\frac{1}{\ln m}$. 

**Step 4.** Calculate the difference coefficient:

(5) $$d_{j}=1-e_{j}.$$

**Step 5.** Calculate the weight of each indicator:

(6) $$W_{j}=\frac{d_{j}}{\sum_{j=1}^{m}d_{j}}.$$

### 4.2. Improved Entropy Method (IEM) and Concrete Calculation Steps

Although the entropy method avoids the deviation caused by human factors, it ignores the importance of the indicators themselves, and the final result often does not accord with the actual situation when dealing with the indicator values with high degree of dispersion, which means the traditional entropy method can be improved [19]. 

Therefore, a judgment matrix based on indicator information entropy is constructed to overcome the problem that too much weight of an indicator will affect the final evaluation result in traditional entropy method. Improved entropy method firstly compares the difference coefficient between indicators in pairs and maps the comparison results to the scales 1–9. Secondly the judgment matrix is obtained and we attempt to calculate the eigenvectors corresponding to the maximum eigenvalues of the matrix, finally we normalize the eigenvectors to obtain the weight of each indicator. 

The weight of each digital economy indicator can be figured out by the following steps based on the improved entropy method:

**Step 1.** Calculate the difference coefficient of each indicator according to the Formulas (2)–(5).

**Step 2.** Calculate the maximum ratio of the difference coefficient:

(7) $$D=\frac{\max d_{j}}{\min d_{j}}\;(j=1,2,\cdots ,m).$$

**Step 3.** Constructed the scale ratio mapped by 1–9:

(8) $$R=\sqrt[{a-1}]{\frac{D}{a}}.$$

In Formula (8), a is the adjustment coefficient and represents the maximum scale value. When D≤9, let a be the integer closest to D, otherwise a is equal to 9. D is assigned to the mapping values from 1 to 9 by calculating the (a−1)th power in Formula (8). The purpose of being divided by a is to make the scales 1~9 in Analytic Hierarchy Process correspond to the mapping values of scales 1~9 in improved entropy method one to one.

**Step 4.** Calculate the mapping values of scales 1–9:

**Step 5.** Construct the judgment matrix R whose element $r_{ij}$ denotes the ratio of the difference coefficient between two indicators:

(9) $$r_{ij}=\frac{d_{i}}{d_{j}}(d_{i}>d_{j}).$$

In this step, since the elements in the judgment matrix R are obtained by Formula (9), the paradox that A is more important than B, B is more important than C, but C is more important than A will not occur. Therefore, the obtained judgment matrix can satisfy the consistency test.

**Step 6.** Calculate the weight $W_{j}$ of each indicator by analytic hierarchy process. After that, digital economy scale score in the i−th year can be obtained as follows:

(10) $$S_{i}=\sum_{j=1}^{n}X_{ij}^{*}\times W_{j}.$$

### 4.3. Comparison of Traditional Entropy Method and Improved Entropy Method

**Figure 1:**
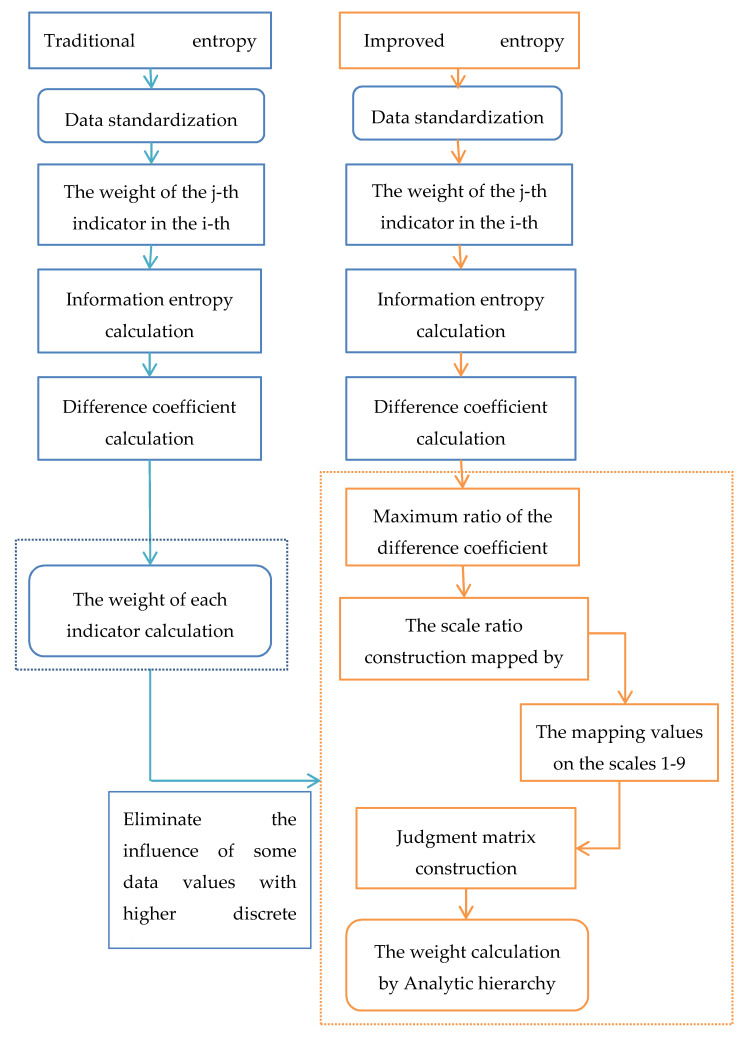
Comparison of traditional entropy method and improved entropy method.

In traditional entropy method, the weight of a single indicator is often too large or too small, so that the single indicator may seriously affect the assessment results. In Figure 1, the improved entropy method can effectively solve this problem. Moreover, it can not only eliminate the influence of some values with a high discrete degree, but also retain the essential characteristics of information entropy.

## 5. Multivariate Statistical Analysis

### 5.1. Principal Component Analysis (PCA) and Concrete Calculation Steps

The purpose of principal component analysis is to obtain a few generality factors mainly by linear combination of multiple indicators and to reduce the dimensions of indicators. In the process of replacing multiple variables with principal components, the loss of variable information should be minimized so that these principal components can synthesize most of the information among the original variables.

The principal component analysis model is as follows:

Let $X={\left(X_{1},X_{2},\cdots ,X_{p}\right)}^{T}$ be a p-dimensional random vector, there exists a linear transformation (11):

(11) $$\left\{ \begin{array}{l} Z_{1}=a_{11}X_{1}+a_{21}X_{2}+\cdots +a_{p1}X_{p},\\ Z_{2}=a_{12}X_{1}+a_{22}X_{2}+\cdots +a_{p2}X_{p},\\ \;\vdots \\ Z_{p}=a_{1p}X_{1}+a_{2p}X_{2}+\cdots +a_{pp}X_{p}, \end{array}\right.$$

and they satisfy the following:

(1) $Z_{i}$ and $Z_{j}$ are independent of each other;
(2) $Var(Z_{1})\geq Var(Z_{2})\geq \cdots \geq Var(Z_{p})$;
(3) $a_{1k}^{2}+a_{2k}^{2}+\cdots +a_{pk}^{2}=1,k=1,2,\cdots ,p.$

Then the i−th principal component of the original variables $X_{1},X_{2},\cdot \cdot \cdot ,X_{p}$ can be denoted as $Z_{i}$. The main calculation steps are as follows:

**Step 1.** Obtain the correlation coefficient matrix Σ of random variable X.

**Step 2.** Calculate the eigenvalue $\lambda_{1}\geq \lambda_{2}\geq \cdots \geq \lambda_{p}\geq 0$ of Σ, then there exists an orthogonal matrix Q such that:

(12) $$Q^{T}\Sigma Q=\Lambda =diag(\lambda_{1},\lambda_{2},\cdots ,\lambda_{p}).$$

Let $Z={\left(Z_{1},Z_{2},\cdots ,Z_{p}\right)}^{T}$, by Formula (11), we can obtain:

(13) $$Z=A^{T}X.$$

Let A=Q, we get:

(14) $$Var(Z)=A^{T}Var(X)A=A^{T}\Sigma A=\Lambda .$$

In this way, conditions (1)–(3) are satisfied. Therefore, it can be known that the linear transformation (11) is orthogonal transformation. Geometrically, the principal component can be obtained by the rotation or reflection of original variable.

Observing (12)–(14), we can know that:

(15) $$\sum_{i=1}^{p}Var(Z_{i})=\lambda_{1}+\lambda_{2}+\cdots +\lambda_{p}=\sigma_{11}+\sigma_{22}+\cdots +\sigma_{pp}.$$

From Formula (15), it can be seen that the sum of variances of original variables is equal to that of principal components. 

**Step 3.** Calculate the contribution rate $\lambda_{i}/\sum_{i=1}^{p}\lambda_{i}$ of the i−th principal component and the accumulative contribution rate $\sum_{i=1}^{m}\lambda_{i}/\sum_{i=1}^{p}\lambda_{i}$. 

When making a principal component analysis, we attempt to use a small number of principal components $Z_{1},Z_{2},\cdots ,Z_{m}\left(m<p\right)$ to replace the original p indicators. Hence, we can choose these m principal components for the next research as long as the cumulative contribution rate of which is greater than or equal to 85%.

**Note:** In practical application, it is difficult for us to obtain the population covariance matrix Σ. Therefore, sample covariance matrix or correlation coefficient matrix is usually used for calculation. Suppose:

(16) $$X=\left[\begin{array}{l} x_{11}\;x_{12}\;\cdots \;x_{1p}\\ x_{21}\;x_{22}\;\cdots \;x_{2p}\\ \;\vdots \;\vdots \;\vdots \\ x_{n1}\;x_{n2}\;\cdots \;x_{np} \end{array}\right],$$

is the sample observation data (p indicators, n samples), $x_{ij}$ is the value of the i−th sample on the j−th indicator. 

**Step 4.** Calculate the load of the principal component. Let S be the sample covariance matrix, then there exists an orthogonal matrix A such that $A^{T}SA=\Lambda$, and

(17) $$Z=\left[\begin{array}{l} z_{11}\;z_{12}\;\cdots \;z_{1p}\\ z_{21}\;z_{22}\;\cdots \;z_{2p}\\ \;\vdots \;\vdots \;\vdots \\ z_{n1}\;z_{n2}\;\cdots \;z_{np} \end{array}\right]=\left[\begin{array}{l} x_{11}\;x_{12}\;\cdots \;x_{1p}\\ x_{21}\;x_{22}\;\cdots \;x_{2p}\\ \;\vdots \;\vdots \;\vdots \\ x_{n1}\;x_{n2}\;\cdots \;x_{np} \end{array}\right]\left[\begin{array}{l} a_{11}\;a_{12}\;\cdots \;a_{1p}\\ a_{21}\;a_{22}\;\cdots \;a_{2p}\\ \;\vdots \;\vdots \;\vdots \\ a_{p1}\;a_{p2}\;\cdots \;a_{pp} \end{array}\right]=XA.$$

A is the load matrix and $z_{ij}$ is the value of the i−th sample on the j−th principal component.

### 5.2. Factor Analysis (FA) and Concrete Calculation Steps

Factor analysis is a statistical technology used to extract common factors by studying the correlation coefficient matrix or covariance matrix of a number of variables, whose core is to reflect most of the information of original variables through a few independent factors.

Factor analysis model can be constructed as follows:

Let $X={\left(X_{1},X_{2},\cdots ,X_{p}\right)}^{T}$ be an observable random vector, and:

(18) $$E\left(X\right)=\mu ={\left(\mu_{1},\mu_{2},\cdots ,\mu_{p}\right)}^{T},\;Var\left(X\right)=\Sigma ={\left(\sigma_{ij}\right)}_{p\times p}.$$

Then the factor analysis model can be obtained as:

(19) $$\left\{ \begin{array}{l} X_{1}-\mu_{1}=a_{11}f_{1}+a_{12}f_{2}+\cdots +a_{1m}f_{m}+\varepsilon_{1}\\ X_{2}-\mu_{2}=a_{21}f_{1}+a_{22}f_{2}+\cdots +a_{2m}f_{m}+\varepsilon_{2}\\ \;\vdots \\ X_{p}-\mu_{p}=a_{p1}f_{1}+a_{p2}f_{2}+\cdots +a_{pm}f_{m}+\varepsilon_{p} \end{array}\right..$$

In Formula (19), $f_{1},f_{2},\cdots ,f_{m}\left(m<p\right)$ are common factors representing the common elements of the original variable and $\varepsilon_{1},\varepsilon_{2}\cdots ,\varepsilon_{m}$ are special factors. Each special factor $\varepsilon_{i}$ only appears in the i−th original variable corresponding to it, and only affects this variable. 

The Model (19) can be transformed into the following form:

(20) X=μ+AF+ε.

In Formula (20), $F={\left(f_{1},f_{2},\cdots ,f_{m}\right)}^{T}$ and $\varepsilon ={\left(\varepsilon_{1},\varepsilon_{2},\cdots ,\varepsilon_{p}\right)}^{T}$ are the vectors whose elements are common factors and special factor respectively, and $A={\left(a_{ij}\right)}_{p\times m}$ is factor load matrix. We assume that:

(21) $$E\left(F\right)=0,\;Var\left(F\right)=I_{m},$$

(22) $$E\left(\varepsilon \right)=0,\;Var\left(\varepsilon \right)=D=diag\left(\sigma_{1}^{2},\sigma_{2}^{2},\cdots ,\sigma_{p}^{2}\right),$$

(23) Cov(F,ε)=0.

From Formulas (21)–(23), we can know that the common factors are uncorrelated to each other, and they form the unit matrix. Any two special factors are uncorrelated, and they have nothing to do with the common factors.

Based on the above discussion on the factor analysis model, we can summarize its main calculation steps as follows:

**Step 1.** Obtain the correlation coefficient matrix.
**Step 2.** Obtain the common factor and load matrix.
**Step 3.** Rotate the load matrix.
**Step 4.** Calculate factor score.

## 6. Numerical Example

According to Section 3, 20 digital economy indicators are selected to construct the indicator evaluation system. In this section, we will choose real data from different departments of the Guangdong Provincial government to verify the improved entropy method, principal component analysis model and factor analysis model.

### 6.1. Data Sources

Based on the data released by China Statistics Bureau, Ministry of Commerce and Guangdong Department of Industry and Information Technology, 20 indicators from 2015 to 2018 shown in Table 2 were selected for the next research. (Data sources: $X_{1}$, $X_{2}$, $X_{3}$, $X_{4}$, $X_{5}$, $X_{6}$, $X_{7}$, $X_{8}$, $X_{9}$, $X_{10}$, $X_{12}$, $X_{13}$, $X_{14}$, $X_{16}$, $X_{18}$, $X_{19}$, $X_{20}$ originate from China Statistical Yearbook. $X_{11}$ originates from the China’s Ministry of Commerce. $X_{15}$ originates from the Department of industry and information technology of Guangdong Province, $X_{17}$ originates from the People’s Government of Guangdong Province).

### 6.2. Analysis of the Development of Digital Economy in Guangdong Province

#### 6.2.1. Analysis Based on the Improved Entropy Method

The difference coefficient of each indicator in Table 1 can be calculated as: 0.2370, 0.5417, 0.3835, 0.2421, 0.2752, 0.3040, 0.3157, 0.3895, 0.2755, 0.2746, 0.5000, 0.2707, 0.4256, 0.3322, 0.2133, 0.2783, 0.2959, 0.2653, 0.2135, 0.3742 by using Formulas (1)–(4) and the data in Table 3. According to Formula (6), the maximum ratio of the difference coefficient is 2.54, so a should be 3 and the R value is 0.92. Therefore, three mapping values can be obtained by calculation. Then we can get Table 4 by the principle of one-to-one correspondence:

The comparison matrix constructed according to Table 4 can be obtained as follows:

$$R=\left[\begin{array}{l} 1.00\;0.33\;0.50\;1.00\;1.00\;1.00\;1.00\;0.50\;1.00\;1.00\;1.00\;1.00\;0.50\;1.00\;1.00\;1.00\;1.00\;1.00\;1.00\;0.50\;\\ 3.00\;1.00\;1.00\;3.00\;2.00\;2.00\;2.00\;1.00\;2.00\;2.00\;1.00\;2.00\;1.00\;2.00\;3.00\;2.00\;2.00\;2.00\;3.00\;2.00\;\\ 2.00\;1.00\;1.00\;2.00\;1.00\;1.00\;1.00\;1.00\;1.00\;1.00\;1.00\;1.00\;1.00\;1.00\;2.00\;1.00\;1.00\;2.00\;2.00\;1.00\;\\ 1.00\;0.33\;0.50\;1.00\;1.00\;1.00\;1.00\;0.50\;1.00\;1.00\;0.50\;1.00\;0.50\;1.00\;1.00\;1.00\;1.00\;1.00\;1.00\;0.50\;\\ 1.00\;0.50\;1.00\;1.00\;1.00\;1.00\;1.00\;1.00\;1.00\;1.00\;0.50\;1.00\;0.50\;1.00\;1.00\;1.00\;1.00\;1.00\;1.00\;1.00\;\\ 1.00\;0.50\;1.00\;1.00\;1.00\;1.00\;1.00\;1.00\;1.00\;1.00\;0.50\;1.00\;1.00\;1.00\;2.00\;1.00\;1.00\;1.00\;2.00\;1.00\;\\ 1.00\;0.50\;1.00\;1.00\;1.00\;1.00\;1.00\;1.00\;1.00\;1.00\;0.50\;1.00\;1.00\;1.00\;2.00\;1.00\;1.00\;1.00\;2.00\;1.00\;\\ 2.00\;1.00\;1.00\;2.00\;1.00\;1.00\;1.00\;1.00\;1.00\;1.00\;1.00\;2.00\;1.00\;1.00\;2.00\;1.00\;1.00\;2.00\;2.00\;1.00\;\\ 1.00\;0.50\;1.00\;1.00\;1.00\;1.00\;1.00\;1.00\;1.00\;1.00\;0.50\;1.00\;0.50\;1.00\;1.00\;1.00\;1.00\;1.00\;1.00\;1.00\;\\ 1.00\;0.50\;1.00\;1.00\;1.00\;1.00\;1.00\;1.00\;1.00\;1.00\;0.50\;1.00\;0.50\;1.00\;1.00\;1.00\;1.00\;1.00\;1.00\;1.00\;\\ 1.00\;1.00\;1.00\;2.00\;2.00\;2.00\;2.00\;1.00\;2.00\;2.00\;1.00\;2.00\;1.00\;2.00\;3.00\;2.00\;2.00\;2.00\;3.00\;1.00\;\\ 1.00\;0.50\;1.00\;1.00\;1.00\;1.00\;1.00\;0.50\;1.00\;1.00\;0.50\;1.00\;0.50\;1.00\;1.00\;1.00\;1.00\;1.00\;1.00\;1.00\;\\ 2.00\;1.00\;1.00\;2.00\;2.00\;1.00\;1.00\;1.00\;2.00\;2.00\;1.00\;2.00\;1.00\;1.00\;2.00\;2.00\;2.00\;2.00\;2.00\;1.00\;\\ 1.00\;0.50\;1.00\;1.00\;1.00\;1.00\;1.00\;1.00\;1.00\;1.00\;0.50\;1.00\;1.00\;1.00\;2.00\;1.00\;1.00\;1.00\;2.00\;1.00\;\\ 1.00\;0.33\;0.50\;1.00\;1.00\;0.50\;0.50\;0.50\;1.00\;1.00\;0.33\;1.00\;0.50\;0.50\;1.00\;1.00\;1.00\;1.00\;1.00\;0.50\;\\ 1.00\;0.50\;1.00\;1.00\;1.00\;1.00\;1.00\;1.00\;1.00\;1.00\;0.50\;1.00\;0.50\;1.00\;1.00\;1.00\;1.00\;1.00\;1.00\;1.00\;\\ 1.00\;0.50\;1.00\;1.00\;1.00\;1.00\;1.00\;1.00\;1.00\;1.00\;0.50\;1.00\;0.50\;1.00\;1.00\;1.00\;1.00\;1.00\;1.00\;1.00\;\\ 1.00\;0.50\;0.50\;1.00\;1.00\;1.00\;1.00\;0.50\;1.00\;1.00\;0.50\;1.00\;0.50\;1.00\;1.00\;1.00\;1.00\;1.00\;1.00\;1.00\;\\ 1.00\;0.33\;0.50\;1.00\;1.00\;0.50\;0.50\;0.50\;1.00\;1.00\;0.33\;1.00\;0.50\;0.50\;1.00\;1.00\;1.00\;1.00\;1.00\;0.50\;\\ 2.00\;0.50\;1.00\;2.00\;1.00\;1.00\;1.00\;1.00\;1.00\;1.00\;1.00\;1.00\;1.00\;1.00\;2.00\;1.00\;1.00\;1.00\;2.00\;1.00\; \end{array}\right]$$

**Figure 2:**
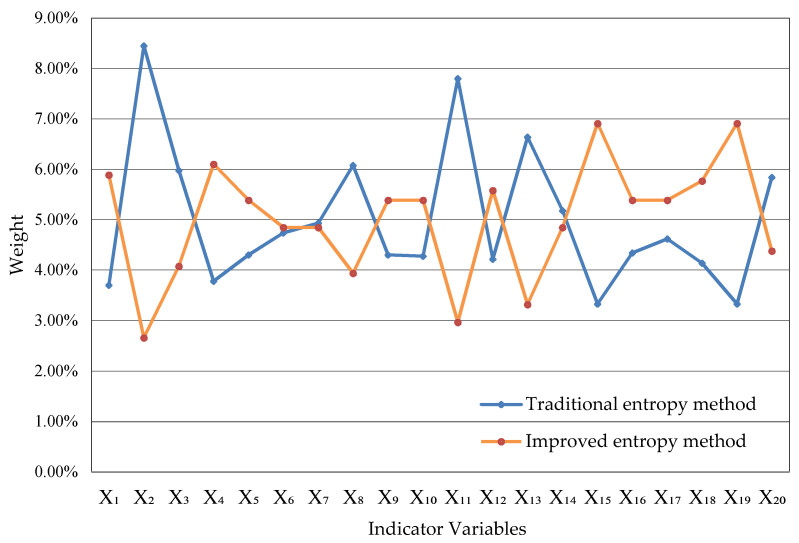
Comparison of each indicator’s weight between traditional entropy and improved entropy method.

Based on the steps in Section 4, we use R software to calculate the weight of each indicator by analytic hierarchy process, that is the improved entropy method. By adding the weights of the corresponding indicators of the four types of digital economy—basic, technology, integration and service types, the proportion of the four types of digital economy in the total scale of digital economy can be obtained. Compared with the traditional entropy method, the results obtained can be shown in Table 5 and Figure 2.

In traditional entropy method, the weight of increase of rural e-commerce comprehensive demonstration counties is 7.80%, but it’s unreasonable because the four-year data of the indicator are 4, 0, 0 and 4. While the weight of increase of rural e-commerce comprehensive demonstration counties calculated by the improved entropy method is 2.97%, which is consistent with the actual situation and indicates that the improved entropy method is better. The weights of four digital economy types can be calculated as shown in Table 6 according to Table 5.

According to Table 5 and Formula (9), the ranking of digital economy in 2015–2018 can be calculated, and the results are shown in Table 7:

Through the weight obtained by the improved entropy method, it can be seen that some of the 20 digital economy indicators have a large difference with the average value, but the weights of most indicators are maintained at about 5%, indicating that the development of digital economy in Guangdong Province was relatively balanced from 2015 to 2018 and will be better in the future. From Table 6, it can be seen that the proportion of the four types of digital economy in the total development of digital economy remains between 23% and 28%, indicating that the four types of digital economy didn’t make a lot of contributions to the digital economy in Guangdong Province from 2015 to 2018, which shows the digital economic types of Guangdong Province are on the road of comprehensive development. In addition, it can be seen from Table 7 that the score of digital economic scale in Guangdong Province has been continuously improving, which indicates that the digital economy of Guangdong Province has been in a state of rapid development, and will continue to do so for a long time in the future.

Nevertheless, we cannot ignore the shortcomings in some areas. According to the improved entropy method, the weight of the increase of rural e-commerce comprehensive demonstration counties in digital economy is only 2.97%, which is 2.03% lower than the average value. This shows that the development of rural e-commerce in Guangdong Province is relatively backward, and there is an obvious digital gap between urban and rural areas. Therefore, we should implement a series of measures to popularize e-commerce in rural areas, stimulate the progress of rural digital economy with the development of information technology industry, and encourage rural areas to use the Internet to promote economic development.

#### 6.2.2. Analysis Based on the Principal Component Analysis Method

**Figure 3:**
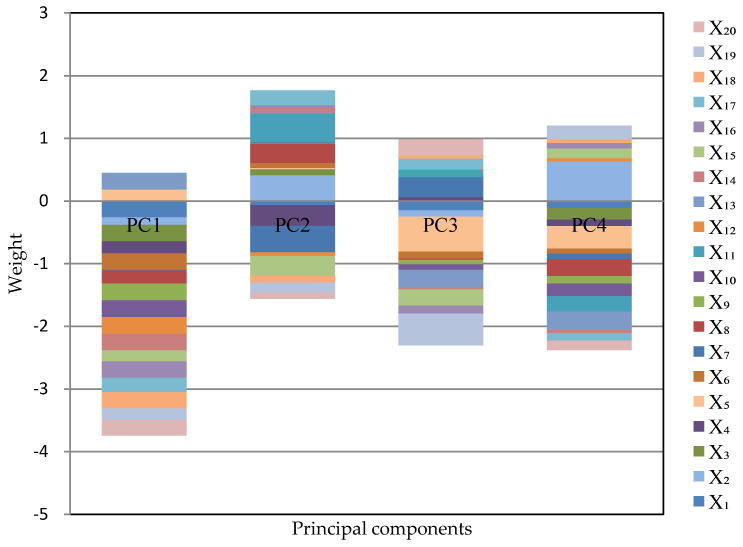
The principle components distribution diagram.

Firstly, we standardized the data in Table 1, and then principal component analysis is performed on the standardized data by R software. Through the observation results, we can obtain the standard deviation, contribution rate and cumulative contribution rate of each principal component, as shown in Table 8:

The load matrix of principal components is shown in Table 9, and we can obtain Figure 3 for more intuitive observation:

It can be seen from Table 8 that in order to achieve the target of dimensionality reduction, we can select the principal components whose cumulative contribution rate reaches 91.91%, namely, PC1 and PC2.

The linear relationships among the first principal component and the original variables are shown in Formula (24).

(24) $$\begin{array}{cc} Z_{1}^{*}= & -0.2618X_{1}-0.1190X_{2}-0.2637X_{3}-0.1900X_{4}+0.1884X_{5}\\ & -0.2628X_{6}-0.0129X_{7}-0.2054X_{8}-0.2680X_{9}-0.2674X_{10}.\\ & +0.0080X_{11}-0.2671X_{12}+0.2521X_{13}-0.2655X_{14}-0.1749X_{15}\\ & -0.2643X_{16}-0.2258X_{17}-0.2616X_{18}-0.1853X_{19}-0.2486X_{20} \end{array}$$

The linear relationships among the second principal component and the original variables are shown in Formula (25).

(25) $$\begin{array}{cc} Z_{2}^{*}= & -0.0644X_{1}+0.4147X_{2}+0.0939X_{3}-0.3297X_{4}+0.0229X_{5}\\ & +0.0782X_{6}-0.4258X_{7}+0.3017X_{8}+0.0109X_{9}+0.0162X_{10}.\\ & +0.4617X_{11}-0.0584X_{12}+0.0112X_{13}+0.0751X_{14}-0.3194X_{15}\\ & +0.0445X_{16}+0.2346X_{17}-0.1056X_{18}-0.1578X_{19}-0.0980X_{20} \end{array}$$

The main component scores in 2015–2018 are shown in Table 10.

We can conclude that: The first two principal components $Z_{1}^{*}$ and $Z_{2}^{*}$ can be selected according to the rule of principal component selection where the cumulative contribution rate should exceed 85%, which can reflect the information of 20 indicators fully. Their expressions are shown in Formulas (24) and (25). The coefficients in Formulas (24) and (25) are called the principal component loading, which represent the correlation coefficient between the principal component and the corresponding original variables. The greater the absolute value of the correlation coefficient, the closer the relationship between the principal component and the variable. Only three coefficients corresponding to the first principal component in Formula (24) are positive, while the other 17 coefficients are negative, which means the first principal component is positively correlated with the number of domain names, the increase of rural e-commerce comprehensive demonstration counties, and the proportion of enterprises with e-commerce transaction activities and is negatively correlated with the other 17 indicators. In this case, the first principal component can be named as overall level factor. Because the second principal component is the difference between the eight indicators--social fixed asset investment in scientific research, social fixed asset investment in scientific research, technical services and the geological prospecting industry, the length of optical cable lines, the number of websites, the embedded system software revenue, the number of designed size enterprises P & D projects, the number of enterprises integrated with industrialization and informatization, the number of terminals in electronic reading room of public library, the number of digital TV users and other indicator variables. Therefore, when the value of the second principal component is close to zero, the values of all indicators are relatively close and can be named as the coordination factors.

All indicator variables are positive in the digital economy evaluation indicator system. Therefore, according to the score of the first principal component, the scores in 2015 and 2016 are higher, which indicate that the development of digital economy in these two years is relatively backward; the scores in 2017 and 2018 are negative, indicating that the development status of digital economy in these two years is good. Generally speaking, from the perspective of the first principal component analysis, the development scale of digital economy in Guangdong Province has been gradually expanding every year in the past four years.

However, from the perspective of the second principal component, although we can see that the absolute value of the scores in 2017 and 2018 is greater than that in 2015 and 2016, indicating that digital economy is progressing steadily. However, from the score of the second principal component in 2015–2018, none of the four years’ score is close to 0, which indicates that there are uncoordinated problems in the development of some fields in these four years from the perspective of the second principal component. Combined with the results of the improved entropy method, it can be seen that the development gaps between some digital economic industries are quite significant. Therefore, in order to make the digital economy develop faster, attention should be paid to the balance of the development in various digital economic fields.

#### 6.2.3. Analysis Based on the Factor Analysis Method

Firstly we standardize the data in Table 2 by SPSS, and the correlation coefficient matrix between 20 indicator variables is calculated, which is used for factor analysis [22]. In the factor analysis, the principal component method is used to extract two common factors, and then the rotation load matrix is obtained by orthogonally rotating the factor load matrix, and the score coefficient matrix can be obtained.

The explained total variance is shown in Table 11.

It can be seen from Table 11 that the cumulative variance contribution rate of the first two common factors reaches 92.071%. Therefore, only two common factors need to be extracted, which can play a good role in dimensionality reduction without losing too much information. Moreover, after the factor rotation, the cumulative contribution rate of the two common factor variances did not change, but the variance of each factor changed slightly, indicating that the factor rotation only reallocated the variance of the two factors, and did not affect the degree of commonality of the original variables. The factor load matrix after rotation is obtained as shown in Table 12.

By observing the load values of the 20 indicators in Table 12 on the two common factors, it can be found that the first common factor mainly explains the 16 indicator variables $X_{1}$, $X_{3}$, $X_{4}$, $X_{5}$, $X_{6}$, $X_{8}$, $X_{9}$, $X_{10}$, $X_{12}$, $X_{13}$, $X_{14}$, $X_{16}$, $X_{17}$, $X_{18}$, $X_{19}$, $X_{20}$. Similarly, it can be seen that the four indicator variables $X_{2}$, $X_{7}$, $X_{11}$, $X_{15}$ can be explained on the second common factor.

**Figure 4:**
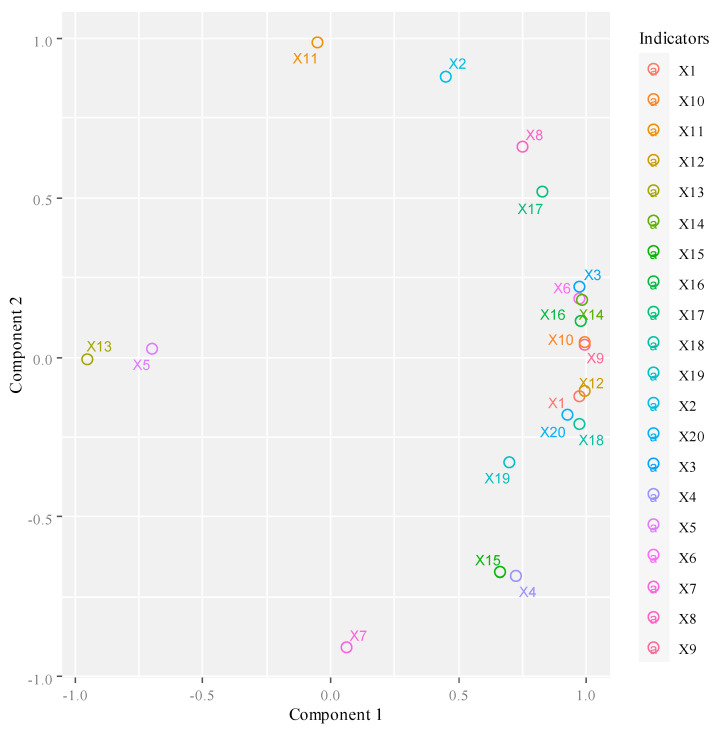
Component graph in rotation space.

The component graph in the rotation space is shown in Figure 4.

As can be seen from Figure 4, $X_{11}$ (increase of rural e-commerce comprehensive demonstration counties), $X_{7}$ (embedded system software revenue), $X_{5}$ (number of domain names), $X_{13}$ (proportion of enterprises with e-commerce transaction activities), $X_{10}$ (social fixed asset investment in information transmission, computer service and software industry) and $X_{9}$ (software business income) are relatively close to the factor axis. Therefore, the information of the four indicators including the proportion of enterprises with e-commerce transaction activities, the number of domain names, information transmission computer services, fixed asset investment of software industry and software business income can be effectively described by the first public factor, while the two indicators of embedded system software income and the increase of rural e-commerce comprehensive demonstration counties are better described by the second public factor. However, if these two common factors are used to describe other indicators, they are not very effective.

**Figure 5:**
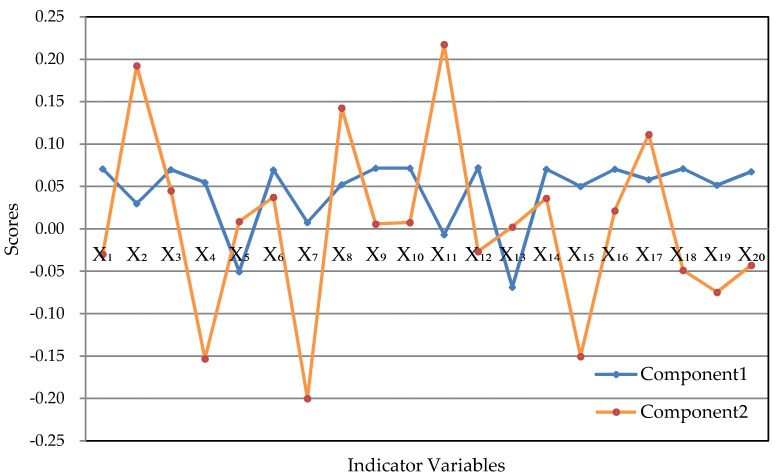
Factor score coefficient graph.

The factor score coefficient matrix is shown in Table 13 and Figure 5.

According to the data in Table 13, the score function of two common factors can be calculated:

(26) $$\left\{ \begin{array}{cc} S_{1} & =0.0708X_{1}+0.0297X_{2}+0.0697X_{3}+0.0547X_{4}-0.0504X_{5}\\ & +0.0696X_{6}+0.0075X_{7}+0.0520X_{8}+0.0717X_{9}+0.0715X_{10}\\ & -0.0068X_{11}+0.0721X_{12}-0.0688X_{13}+0.0704X_{14}+0.0501X_{15}\\ & +0.0704X_{16}+0.0581X_{17}+0.0711X_{18}+0.0515X_{19}+0.0675X_{20}\\ S_{1} & =-0.0297X_{1}+0.1924X_{2}+0.0452X_{3}-0.1531X_{4}+0.0087X_{5}\\ & +0.0375X_{6}-0.1999X_{7}+0.1427X_{8}+0.0059X_{9}+0.0077X_{10}\\ & +0.2176X_{11}-0.0266X_{12}-0.0022X_{13}+0.0362X_{14}-0.1503X_{15}\\ & +0.0216X_{16}+0.1114X_{17}-0.0488X_{18}-0.0746X_{19}-0.0427X_{20} \end{array}\right..$$

In combination with Formula (26) and the variance contribution rate of each common factor in Table 11, the development of digital economy from 2015 to 2018 can be evaluated and scored. The scoring model is:

(27) $$S=\frac{69.339S_{1}+22.732S_{2}}{92.071}.$$

According to the scoring model obtained by Formula (27), the comprehensive score of each year from 2015 to 2018 is calculated, and the chart of digital economic scores of Guangdong Province in four years is drawn, as shown in Figure 3.

**Figure 6:**
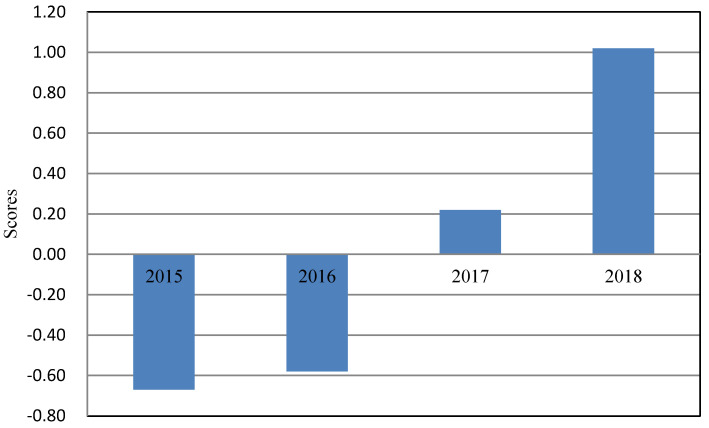
Chart of digital economic scores.

It can be seen from Figure 6 that the score of digital economy in Guangdong Province is getting higher and higher from 2015 to 2018, indicating that the development of digital economy in Guangdong Province is progressing every year. Therefore, the development situation of digital economy in Guangdong Province will be better in the future.

### 6.3. Comparison of IEM, PCA and FA

In this paper, we mainly took advantage of three methods (IEM, PCA and FA) to evaluate the overall development scale of digital economy in Guangdong Province from 2015 to 2018 under the indicator evaluation system. Some corresponding conclusions from different perspectives were put forward in each method. 

In order to make our work clear, we briefly summarize and compare the general method, calculation steps and main results of each method in Table 14. The various conclusions of this paper can be taken into consideration by decision makers to help them develop strategies according to their different requirements.

## 7. Conclusions and Suggestion

Through the establishment of digital economy indicator evaluation system, this paper conducts quantitative analysis on 20 indicator variables which can be divided into four digital economic types in Guangdong Province from 2015 to 2018. Firstly, the weight of each indicator was calculated by the improved entropy method, and then the proportion of the four economic types was calculated. It was concluded that the overall development of each digital field in Guangdong Province was well, but some digital economic fields also had poor development. Secondly, using the method of principal component analysis to compress the information of 20 indicators, and finally two principal components which can be expressed as a linear combination of these 20 indicators were obtained. On this basis, through the discussion and analysis of the scale of digital economy in Guangdong Province from 2015 to 2018, it can be found that although the development trend of digital economy was better year by year, there are also unbalanced development problems. Finally, factor analysis was used to reduce dimension and two common factors were obtained, which effectively reduced the number of indicators to be analyzed and retained most of the information of the original indicators, thus improving the efficiency of studying the development of digital economy in Guangdong Province. In addition, a scoring model was established through factor analysis. It was found that the comprehensive score of each year was increasing, which showed that the development prospect of digital economy in Guangdong is optimistic.

In the future study, we will combine these methods in this paper with some algorithms in machine learning, such as regression analysis and cluster analysis, and continue to study the development of digital economy from different perspectives and provide more practical conclusions.

## Figures and Tables

**Table 1:** The selected digital economy indicators in evaluation system.

Indicator Category	Indicator Variables	Indicator Names	Unit
Basic-Type Digital Economy	$X_{1}$	Length of optical cable line	km
	$X_{2}$	Telephone penetration rates	set/person
	$X_{3}$	Number of Internet broadband users	ten thousand
	$X_{4}$	Number of websites	ten thousand
	$X_{5}$	Number of domain names	ten thousand
Technology-Type Digital Economy	$X_{6}$	IT service revenue	ten thousand yuan
	$X_{7}$	Embedded system software revenue	ten thousand yuan
	$X_{8}$	Total telecom services	100 million yuan
	$X_{9}$	Software business income	ten thousand yuan
	$X_{10}$	Social fixed asset investment in information transmission, computer service and software industry	100 million yuan
Integration-Type Digital Economy	$X_{11}$	Increase of rural e-commerce comprehensive demonstration counties	unit/year
	$X_{12}$	Number of designed size enterprises P & D projects	unit
	$X_{13}$	Proportion of enterprises with e-commerce transaction activities	%
	$X_{14}$	Number of enterprises with informatization	unit
	$X_{15}$	Number of enterprises integrated with industrialization and informatization	unit
Service- Type Digital Economy	$X_{16}$	E-commerce turnover	100 million yuan
	$X_{17}$	Number of public information on government websites	piece
	$X_{18}$	Number of terminals in electronic reading room of public library	set
	$X_{19}$	Number of digital TV users	ten thousand households
	$X_{20}$	Social fixed asset investment in scientific research, technical services and the geological prospecting industry	100 million yuan

**Table 2:** Correspondence between scale and mapping value.

**Scales**	1	2	3	4	5	6	7	8	9
**Mapping Values**	$1\times R^{0}$	$2\times R^{1}$	$3\times R^{2}$	$4\times R^{3}$	$5\times R^{4}$	$6\times R^{5}$	$7\times R^{6}$	$8\times R^{7}$	$9\times R^{8}$

**Table 3:** The concrete values corresponding to the indicators in Guangdong Province from 2015 to 2018.

Indicator Variables	2018	2017	2016	2015
$X_{1}$	2,588,927.29	2,408,413.54	2,101,665.08	1,645,703.16
$X_{2}$	167.76	154.02	154.18	159.34
$X_{3}$	3597.80	3246.80	2779.40	2682.70
$X_{4}$	72.76	77.75	72.82	67.10
$X_{5}$	449.03	397.87	556.57	497.10
$X_{6}$	62,255,565.60	49,198,710.10	38,958,597.30	31,290,890.50
$X_{7}$	21,423,301.00	25,993,356.50	23663145.20	22,523,703.20
$X_{8}$	7798.43	3579.70	1991.31	3150.03
$X_{9}$	106,874,315.50	96,812,074.50	82,233,914.90	71,051,485.20
$X_{10}$	569.22	541.92	506.72	477.81
$X_{11}$	4.00	0.00	0.00	4.00
$X_{12}$	76,985.00	73,439.00	50,740.00	37,375.00
$X_{13}$	9.80	9.70	11.60	11.50
$X_{14}$	124,606.00	113,151.00	99,568.00	94,003.00
$X_{15}$	74.00	82.00	79.00	52.00
$X_{16}$	44,934.50	37,095.90	30,449.80	23,891.60
$X_{17}$	2,870,056.00	2,624,963.00	2,340,155.00	2,487,318.00
$X_{18}$	10,847.00	10,928.00	9723.00	9034.00
$X_{19}$	1760.70	1691.66	1755.87	1487.43
$X_{20}$	256.52	266.82	223.49	218.14

**Table 4:** Correspondence between scale and mapping value.

Scales	1	2	3
**Mapping Values**	1	1.84	2.54

**Table 5:** Comparison of each indicator’s weight between traditional entropy and improved entropy method.

Indicator Variables	Traditional Entropy	Improved Entropy	Indicator Variables	Traditional Entropy	Improved Entropy
$X_{1}$	3.70%	5.89%	$X_{11}$	7.80%	2.97%
$X_{2}$	8.45%	2.66%	$X_{12}$	4.22%	5.58%
$X_{3}$	5.98%	4.08%	$X_{13}$	6.64%	3.32%
$X_{4}$	3.78%	6.10%	$X_{14}$	5.18%	4.85%
$X_{5}$	4.30%	5.39%	$X_{15}$	3.33%	6.91%
$X_{6}$	4.74%	4.85%	$X_{16}$	4.34%	5.39%
$X_{7}$	4.93%	4.85%	$X_{17}$	4.62%	5.39%
$X_{8}$	6.08%	3.94%	$X_{18}$	4.14%	5.77%
$X_{9}$	4.30%	5.39%	$X_{19}$	3.33%	6.91%
$X_{10}$	4.28%	5.39%	$X_{20}$	5.84%	4.38%

**Table 6:** Proportion of four digital economy types in improved entropy method.

Economic Types	Basic-Type	Technology-Type	Integration-Type	Service-Type
Improved Entropy Method	24.12%	24.42%	23.63%	27.84%

**Table 7:** Scale scores of digital economy in 2015–2018.

Year	Scale Scores of Digital Economy
2015	0.1397
2016	0.4229
2017	0.6639
2018	0.8249

**Table 8:** Standard deviation, contribution rate and cumulative contribution rate of principal components.

Principal Component	PC1	PC2	PC3	PC4
Standard Deviation	3.7143	2.1413	1.2722	0.0000
Proportion of Variance	0.6898	0.2293	0.0809	0.0000
Cumulative Proportion	0.6898	0.9191	1.0000	1.0000

**Table 9:** The load matrix.

Indicator Variables	PC1	PC2	PC3	PC4
$X_{1}$	−0.2618	−0.0644	−0.1472	−0.1070
$X_{2}$	−0.1190	0.4147	−0.1003	0.6332
$X_{3}$	−0.2637	0.0939	0.0148	−0.1876
$X_{4}$	−0.1900	−0.3297	0.0468	−0.1056
$X_{5}$	0.1884	0.0229	−0.5603	−0.3591
$X_{6}$	−0.2628	0.0782	−0.1093	−0.0803
$X_{7}$	−0.0129	−0.4258	0.3207	−0.0858
$X_{8}$	−0.2054	0.3017	−0.0210	−0.2707
$X_{9}$	−0.2680	0.0109	−0.0741	−0.1225
$X_{10}$	−0.2674	0.0162	−0.0877	−0.1981
$X_{11}$	0.0080	0.4617	0.1155	−0.2465
$X_{12}$	−0.2671	−0.0584	0.0145	0.0535
$X_{13}$	0.2521	0.0112	−0.2753	−0.2939
$X_{14}$	−0.2655	0.0751	−0.0329	−0.0496
$X_{15}$	−0.1749	−0.3194	−0.2608	0.1546
$X_{16}$	−0.2643	0.0445	−0.1290	0.0873
$X_{17}$	−0.2258	0.2346	0.1657	−0.1189
$X_{18}$	−0.2616	−0.1056	0.0530	0.0637
$X_{19}$	−0.1853	−0.1578	−0.5046	0.2124
$X_{20}$	−0.2486	−0.0980	0.2523	−0.1541

**Table 10:** Principal component scores from 2015 to 2018.

Year	PC1	PC2	PC3	PC4
2018	−3.9305	2.0809	−0.5484	0.0000
2017	−2.2090	−2.1390	1.2060	0.0000
2016	2.0177	−1.5280	−1.5297	0.0000
2015	4.1218	1.5860	0.8722	0.0000

**Table 11:** Explained total variance.

Components	Initial Eigenvalue			Extraction Sum of Squares		
	Total	Variance %	Accumulate %	Total	Variance %	Accumulate %
1	13.8680	69.3390	69.3390	13.8680	69.3390	69.3390
2	4.5460	22.7320	92.0710	4.5460	22.7320	92.0710
3	1.5860	7.9290	100.0000	1.5860	7.9290	100.0000
4	0.0000	0.0000	100.0000	0.0000	0.0000	100.0000
5	0.0000	0.0000	100.0000	**Rotation Sum of Squares**		
6	0.0000	0.0000	100.0000	**Total**	**Variance %**	**Accumulate %**
7	0.0000	0.0000	100.0000	7.7510	38.7570	38.7570
8	0.0000	0.0000	100.0000	7.5900	37.9500	76.7070
9	0.0000	0.0000	100.0000	4.6590	23.2930	100.0000
10	0.0000	0.0000	100.0000	0.0000	0.0000	100.0000
……	……	……	……	—	—	—
20	0.0000	0.0000	100.0000	—	—	—

**Table 12:** Rotational component matrix.

Indicator Variables	Components		Indicator Variables	Components	
	1	2		1	2
$X_{1}$	0.9759	−0.1205	$X_{11}$	−0.0491	0.9888
$X_{2}$	0.4519	0.8819	$X_{12}$	0.9941	−0.1061
$X_{3}$	0.9753	0.2199	$X_{13}$	−0.9531	−0.0041
$X_{4}$	0.7263	−0.6853	$X_{14}$	0.9831	0.1791
$X_{5}$	−0.6963	0.0291	$X_{15}$	0.664	−0.6735
$X_{6}$	0.9733	0.1850	$X_{16}$	0.9806	0.1130
$X_{7}$	0.0631	−0.9084	$X_{17}$	0.828	0.5192
$X_{8}$	0.7507	0.6601	$X_{18}$	0.9755	−0.2074
$X_{9}$	0.9951	0.0415	$X_{19}$	0.6982	−0.3287
$X_{10}$	0.9929	0.0500	$X_{20}$	0.9272	−0.1804

**Table 13:** Factor score coefficient table.

Indicator Variables	Components		Indicator Variables	Components	
	1	2		1	2
$X_{1}$	0.0708	−0.0297	$X_{11}$	−0.0068	0.2176
$X_{2}$	0.0297	0.1924	$X_{12}$	0.0721	−0.0266
$X_{3}$	0.0697	0.0452	$X_{13}$	−0.0688	0.0022
$X_{4}$	0.0547	−0.1531	$X_{14}$	0.0704	0.0362
$X_{5}$	−0.0504	0.0087	$X_{15}$	0.0501	−0.1503
$X_{6}$	0.0696	0.0375	$X_{16}$	0.0704	0.0216
$X_{7}$	0.0075	−0.1999	$X_{17}$	0.0581	0.1114
$X_{8}$	0.0520	0.1427	$X_{18}$	0.0711	−0.0488
$X_{9}$	0.0717	0.0059	$X_{19}$	0.0515	−0.0746
$X_{10}$	0.0715	0.0077	$X_{20}$	0.0675	−0.0427

**Table 14:** Comparison of IEM, PCA and FA.

Methods	Improved Entropy Method (IEM)	Principal Component Analysis (PCA)	Factor Analysis (FA)
General Method	Compared with the traditional entropy method, IEM draws on the empowerment idea of Analytic Hierarchy Process.	Obtain a few representative factors by linear combination of multiple indicators.	Use a few factors to describe the relationship among indicators
Calculation Steps	Step 1: Calculate the difference coefficient of each indicator; Step 2: Calculate the maximum ratio of the difference coefficient; Step 3: Construct the scale ratio mapped by 1–9; Step 4: Calculate the mapping values of scales 1–9; Step 5: Construct the judgment matrix *R*; Step 6: Calculate the weight of each indicator by AHP (analytic hierarchy process).	Step 1: Obtain the correlation coefficient matrix; Step 2: Calculate the eigenvalues and eigenvectors; Step 3: Calculate the principal component contribution rate and the accumulative contribution rate; Step 4: Calculate the load of the principal component.	Step 1: Obtain the correlation coefficient matrix; Step 2: Obtain the common factor and load matrix; Step 3. Rotate the load matrix; Step 4. Calculate factor score.
Main Results	Obtain the weight of each indicator. Evaluate the overall development scale of digital economies.	Obtain the concrete expressions of the two principal components. Analyze the coefficients of some indicators.	Obtain the concrete expressions of the two common factors. Present the scores of digital economies.
